# Growing early career researchers pathways through co-review

**DOI:** 10.1038/s41467-024-45269-0

**Published:** 2024-03-05

## Abstract

To build pathways to constructive and engaging peer review for the next generation of scientists, we invite all our reviewers to co-review with an Early Career Researcher in their group and let us know. We will ensure ECRs are recognised for their contribution.

N*ature Communications* can look back on three successful peer review mentoring programs, run in 2020, 2021 and 2022. These initiatives were aimed at providing early career researchers (ECRs), mainly PhD students and postdoctoral researchers, hands-on training in peer review. The program, organised by the journal’s ECR working group, featured webinars explaining the importance of peer review as well as the underlying workflows and editorial processes, while also providing practical guidance to drafting a constructive report, aimed at helping authors through the process rather than being a barrier to publication.

A subset of mentees were subsequently paired with editors of *Nature Communications* and other journals based on their research expertise and invited to review a suitable manuscript under consideration at the journal, putting into practice what they had learned during the webinars. After finishing the review, the ECRs received customised feedback from the editors, including suggestions for improvement and general tips, while also gaining appropriate reviewer credit for their contribution. Similar to the regular peer review process, the ECRs received the reports from the other established reviewers further providing insights into the evaluation of manuscripts and the peer review process.

Feedback from these programs has been overwhelmingly positive. The programs have grown consistently in size from 30 ECRs participating in the hands-on phase in 2020 (90 participating in webinars) to enrolling more than 200 in 2021 and 2022 (over 1000 participating in the webinars in total). We are confident that ECRs have gained important practical skills that will help them draft future reviewer reports, in addition to improving their confidence and supporting their professional development. Their reports have also helped with editorial decisions in over 80% of the cases and we are committed to further interact with those ECR reviewers in the coming years as they progress in their careers.

*Nature Communications* aims to further expand the involvement of young researchers in the peer review process and to provide them with an opportunity to have their reviewer activity acknowledged.

“*Nature Communications* aims to further expand the involvement of young researchers in the peer review process and to provide them with an opportunity to have their reviewer activity acknowledged”

In the meantime, we have been inspired by the co-review mentoring initiative, launched by a subset of Reviews journals within the nature portfolio in 2020, which we are rolling out to the whole journal this week. The co-review initiative encourages experienced researchers to involve an ECR in the peer review process and provides information on available optional online training. While the joint report is submitted by the established reviewer, an account is created the ECR in our online submission system.

This initiative ensures that the ECRs’ contribution to the peer review process is properly credited. ECRs are most likely directly involved in the experimental part of the research, rendering their technical feedback comprehensive and valuable. Furthermore, by performing the peer review process alongside an experienced researcher who will guide their critical assessment of the study and emphasise clear, effective and constructive communication, the ECR will learn and hone their skills, preparing them for independent review in the future.

A group of 25 *Nature Communications* editors have been running a small pilot version of the program since May 2023 and have seen that around 25% of the established reviewers that accept to review take up the offer of involving an ECR. Given the encouraging uptake rate and positive feedback, we are now expanding this initiative to the whole journal.

If you are an ECR wanting to get further involved in peer review, we suggest you speak to your supervisor/PI/group leader to let them know you would be interested in our co-review scheme. Do not hesitate to then contact the ECR working group (naturecommsecr@nature.com) directly so that we know to contact you both if a manuscript in your field is under consideration. We hope our programs will help train a young generation of diverse researchers, (representing the future of research), who will have the knowledge and first-hand experience of peer review to help shape its future.

